# Dementia in Scottish military veterans: early evidence from a retrospective cohort study

**DOI:** 10.1017/S0033291721002440

**Published:** 2023-02

**Authors:** B. P. Bergman, D. F. Mackay, J. P. Pell

**Affiliations:** Institute of Health and Wellbeing, University of Glasgow, Glasgow G12 8RZ, UK

**Keywords:** Dementia, military veterans, mood disorder, post-traumatic stress disorder, retrospective cohort studies

## Abstract

**Background:**

Few studies have examined whether UK military veterans are at an increased risk of dementia. We explored the risk of dementia in Scottish military veterans aged up to 73 years in comparison with people who have never served.

**Methods:**

Retrospective cohort study of 78 000 veterans and 253 000 people with no record of service, matched for age, sex and area of residence, with up to 37 years follow-up, using Cox proportional hazard analysis to compare risk of dementia in veterans and non-veterans, overall and by subgroup.

**Results:**

Dementia was recorded in 0.2% of both veterans and non-veterans overall, Cox proportional hazard ratio 0.98, 95% confidence interval (CI) 0.82–1.19, *p* = 0.879 (landmark age: 50 years), with no difference for men but increased risk in veteran women and Early Service Leavers. Post-traumatic stress disorder (PTSD) was associated with a higher risk of dementia in both veterans and non-veterans, although possibly to a lesser degree in veterans. A history of mood disorder was strongly associated with developing dementia, greater in veterans than in non-veterans, odds ratio 1.54, 95% CI 1.01–2.35, *p* = 0.045.

**Conclusions:**

There was no evidence to suggest that military service increased the risk of dementia, although this may change as the cohort ages. The well-documented association with PTSD shows no evidence of being stronger in veterans; by contrast, the association of mood disorder with dementia is much stronger in veterans. Healthcare providers should carefully assess the cognitive status of older veterans presenting with depressive illness in order to identify early dementia and ensure optimum management.

## Introduction

The reduction in premature mortality in the latter half of the 21st century has paradoxically created a new challenge; more people than ever are living with dementia, creating a major burden on health and social care. The term ‘dementia’ covers a number of clinical entities of which the most common are Alzheimer's disease, diffuse Lewy body dementia, fronto-temporal dementia and vascular dementia (Bolla, Filley, & Palmer, 2000). In this paper, all subtypes will be considered under the single term ‘dementia’. In 2015, an estimated 47 million people were living with dementia worldwide, incurring a cost to society of some US$818 billion, and the prevalence has been projected to triple by 2050 (Livingston et al., 2017). In the UK, around 7.1% of people aged over 65 years are living with dementia, but the prevalence rises steeply with age and the condition affects 30% of those aged 90–94 years (Prince et al., 2014). Dementia shares many risk factors with cardiovascular disease, including hypertension, hypercholesterolaemia, obesity and diabetes (Kivipelto et al., 2006), together with poor educational attainment (Sharp & Gatz, 2011), while age-associated health decline and frailty also increase risk (Song, Mitnitski, & Rockwood, 2011). It is unsurprising, in view of the association with cardiovascular risk factors, than a positive association with inflammatory cytokines has been described (Angelopoulos et al., 2008). There is some evidence that the incidence may be stabilising or declining, especially in Western high-income countries (Roehr, Pabst, Luck, & Riedel-Heller, 2018), which may be associated with improvements in education and control of cardiovascular risk factors (Prince et al., 2014).

In the USA, studies have shown an increased prevalence of dementia in veterans in association with post-traumatic stress disorder (PTSD) (Qureshi et al., 2010; Yaffe et al., 2010), traumatic and mild traumatic brain injury (Barnes et al., 2014; 2018), having been a prisoner of war (Meziab et al., 2014), and depression (Byers, Covinsky, Barnes, & Yaffe, 2012; Rafferty, Cawkill, Stevelink, Greenberg, & Greenberg, 2018). However, none of these studies compared veterans with non-veterans. There is a paucity of studies on the risk of dementia in UK veterans apart from a recent case-control study (Greenberg, Rafferty, Greenberg, & McKenzie, 2020), and Rafferty et al. (2018) have recommended a large-scale cohort study to examine the association between mental health conditions and dementia in a UK military veteran population. Dementia may have a vascular aetiology, and our earlier studies (Bergman, Mackay, & Pell, 2014, 2018) have shown an increased risk of cardiovascular disease in UK veterans, also suggesting that they may be at an increased risk of dementia. An understanding of whether the risk of dementia in veterans differs from the wider community is of importance in planning care and welfare services for an ageing veteran population, and in this study we aim to contribute to filling this knowledge gap. We used data from a large retrospective cohort study of military veterans in Scotland to examine the risk of dementia in comparison with people with no record of military service, and to explore associations with mental health diagnoses.

## Methods

We examined data from the Trends in Scottish Veterans' Health study, a retrospective cohort study of all 78 385 military veterans resident in Scotland who met the eligibility criteria, and a comparison group of 252 637 individuals with no record of service matched 3:1 for age, sex and postcode sector of residence (mean population: 5000). Veterans were eligible for inclusion if they were born between 1 January 1945 and 31 December 1995 and were registered with National Health Service (NHS) Scotland both before and after service. The study follows on from the Scottish Veterans Health Study, using similar methodology which is described elsewhere (Bergman et al., 2014). Demographic data obtained from electronic NHS registration records were linked at an individual level to routine hospital admissions data (Scottish Morbidity Record SMR01), psychiatric hospital admissions and day care records (SMR04), and death certificates to provide information on inpatient diagnoses of dementia, PTSD, mood (depressive) disorder and all-cause death. Dates of entering and leaving the service, for veterans, were obtained from the Scottish NHS registration record. The maximum period of follow-up was from 1 January 1981 (or date of leaving the Armed Forces, for veterans, if later) to 31 December 2017. The data extract was pseudo-anonymised and approval for the study was granted by the Public Benefit and Privacy Panel of the Information Services Division of NHS Scotland, approval number 1718-0133. As this was a secondary data study, individual consent was not required.

A measure of socio-economic status (SES) is provided by the Scottish Index of Multiple Deprivation (SIMD), which is based on 6505 data zones, derived from postcode of residence, with a mean population of 800. Deprivation status is calculated from information on income, employment, health, education (including skills and training), housing, crime, and access to services. The SIMD has been used to derive quintiles of SES for the Scottish population; ranging from 1 (most deprived) to 5 (least deprived) (Scottish Government, 2012). The cohort participants were categorised according to these quintiles using postcode of residence. The diagnosis of dementia was classified as ICD-10 G30 or F00–F03, or ICD-9 290 or 331.0, at any position in the SMR record. Severe stress or PTSD was defined as ICD-10 F43, or ICD-9 300 or 308–309. Mood disorder was defined as ICD-10 F30–F39, or ICD-9 296. ‘Early Service Leavers’ (ESL) were defined as veterans who had left with less than 3 years' service. Although shorter than the current 4-year minimum, this ensured that veterans who completed the earlier minimum of 3 years' service were not incorrectly classified as ESL (Bergman, Mackay, Smith, & Pell, 2016).

### Statistical methods

Cox proportional hazard models were used to examine the association between veteran status and cumulative risk of dementia, overall and in association with specific mental health conditions, using age as the time-dependent variable, age at first record of dementia as the failure time, and death (if no dementia) as the censor time (Thiébaut & Bénichou, 2004). Hazard ratios (HRs) and *p* values were calculated and the *a priori* rejection level was set at 0.05. Proportionality was tested using methodology based on Schoenfeld residuals (Grambsch & Therneau, 1994). The models were run univariably and then repeated adjusting for the potential confounding effect of SES. The analyses were repeated stratifying by 5-year bands of birth year, to examine birth cohort effects, and stratifying by length of service. Collinearity of variables with veteran status was excluded in the analysis. Odds ratios (ORs) were used to examine the risk of mental health comorbidities or antecedent diagnoses. All analyses were performed using Stata^®^ v.16.

## Results

After data cleansing to remove incomplete or invalid records, 78 157 (99.7%) veterans and 252 637 (100%) non-veterans were included in the analyses. There were 70 581 (90.3%) male veterans and 7573 (9.7%) female, in accordance with the gender balance of the armed forces. The mean period of follow-up was 32.5 years, and there were a total of 10.6 million person-years of follow-up among veterans and non-veterans combined. The oldest cohort members were 73 years of age at the end of follow-up.

**Fig. 1.**
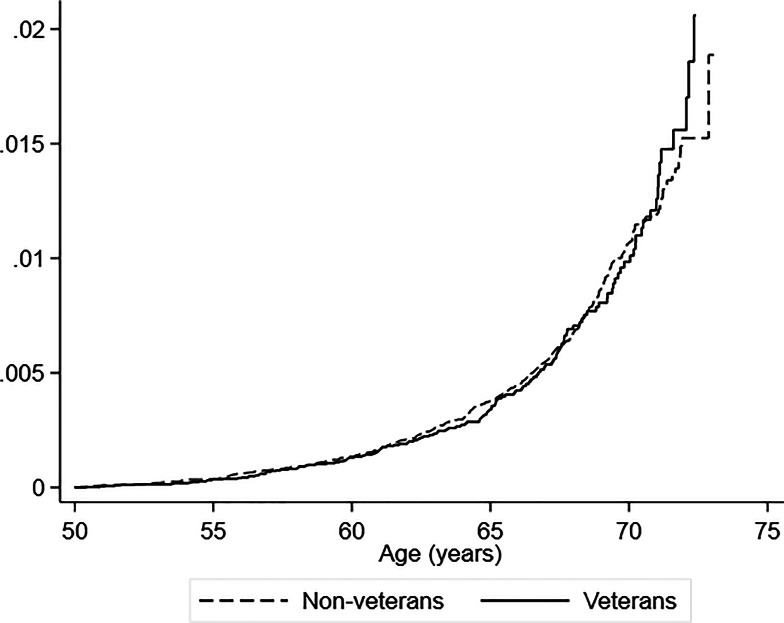
Nelson–Aalen plot of risk of dementia by veteran status.

By the end of follow-up, 154 (0.20%) veterans had a record of a dementia diagnosis, compared with 490 (0.20%) non-veterans (Table 1). Analysed using a landmark age of 50 years, there was no statistically significant difference in risk between veterans and non-veterans, Cox proportional HR 0.98, 95% confidence interval (CI) 0.82–1.19, *p* = 0.879. The Nelson–Aalen cumulative hazard plot is shown in Fig. 1, demonstrating no overall difference between veterans and non-veterans. There was no increase in risk in veterans among men, HR 0.93, 95% CI 0.76–1.14, *p* = 0.481. There was an increased risk for veteran women which almost achieved statistical significance, HR 1.76, 95% CI 0.95–3.23, *p* = 0.070, although the number of cases was small.

**Table 1.** Cox proportional HRs for dementia in veterans, overall and by subgroup, referent to all non-veterans

	Veterans *n* = 78 157	Non-veterans *n* = 252 637	Univariable			Multivariable^a^		
	Cases	Cases	HR	95% CI	*p*	HR	95% CI	*p*
Overall	154	490	0.98	0.82–1.19	0.879	0.95	0.79–1.15	0.622
Men	^b^	^b^	0.93	0.76–1.14	0.481	0.90	0.74–1.10	0.297
Women	^b^	^b^	1.76	0.95–3.23	0.070	1.73	0.94–3.18	0.076
ESL	^b^	^b^	1.29	0.95–1.75	0.117	1.18	0.87–1.61	0.276
Non-ESL	^b^	^b^	0.88	0.70–1.11	0.286	0.87	0.70–1.10	0.244

HR, hazard ratio; CI, confidence interval; ESL, Early Service Leavers.

a Adjusted for socioeconomic status (SIMD quintile).

b Omitted due to small numbers in some cells.

**Fig. 2.**
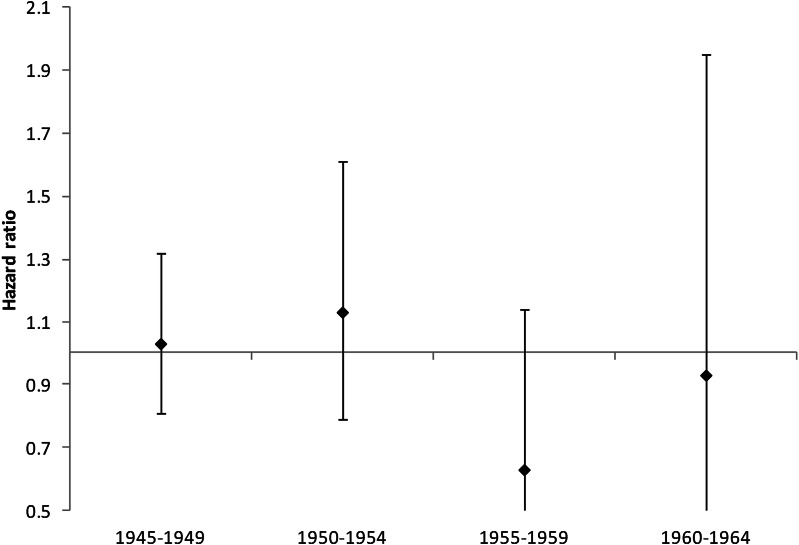
HRs for risk of dementia by birth cohort, veterans compared with non-veterans. Error bars represent 95% CIs.

There was no significant impact of length of service. Among veterans, ESL had a non-significantly increased risk of dementia when compared with all non-veterans, which reduced after adjusting for SES (Table 1). Comparison between ESL and those who completed at least the minimum military engagement confirmed a statistically significant increased risk in this group, HR 1.46, 95% CI 1.03–2.09, *p* = 0.040. Controlling for SES only slightly reduced the excess risk in ESL, HR 1.41, 95% CI 0.99–2.03, *p* = 0.059, but it was attenuated after adjusting for mood disorder, HR 1.31, 95% CI 0.92–1.87, *p* = 0.138. Analysis of HR by birth cohort showed no significant difference for any group (Fig. 2).

By the end of follow-up, 65 (42.2%) of the veterans with dementia had died, compared with 254 (51.8%) of the non-veterans. The difference was statistically significant, OR 0.81, 95% CI 0.66–1.00, *p* = 0.037. The majority of deaths in non-veterans were coded as due to dementia, including Alzheimer's disease and Parkinson's disease, although a small number were due to congenital conditions which would have precluded military service. There was no predominant cause of death among the veterans with dementia.

A diagnosis of PTSD at any time increased the likelihood of developing dementia in both veterans and non-veterans, but to a greater extent in non-veterans. For non-veterans, there was a fourfold increase in risk. A total of 0.78% of those with a record of PTSD developed dementia, compared with 0.19% of those without PTSD, OR 4.13, 95% CI 2.47–6.89, *p* < 0.001. For veterans, the increase in risk was only threefold; 0.57% of those with a record of PTSD developed dementia compared with 0.19% of those without PTSD, OR 2.98, 95% CI 1.32–6.72, *p* = 0.006. Among those with a PTSD diagnosis, we found no evidence that veterans were at an increased risk of developing dementia compared with non-veterans, OR 0.73, 95% CI 0.28–1.88, *p* = 0.511. Testing for interaction was non-significant, *p* = 0.411. In all veteran cases, and the majority of non-veterans, the PTSD diagnosis preceded diagnosis of dementia, although in a small number of cases the interval between diagnoses was short, raising the possibility of the early symptoms of dementia having been misclassified as PTSD.

Mood disorder was also strongly associated with dementia, the diagnosis of mood disorder predating dementia, sometimes by many years, in the majority of cases. Of veterans who had a record of mood disorder at any time, 1.36% developed dementia, compared with 0.16% of those without mood disorder, OR 8.43, 95% CI 5.72–12.42, *p* < 0.001. The increase in risk was lower in non-veterans; 0.88% of those with a history of mood disorder were diagnosed with dementia, compared with 0.17% of those without a record of mood disorder, OR 5.07, 95% CI 3.90–6.60, *p* < 0.001. Among those with a record of mood disorder, the risk of dementia was significantly higher in veterans compared with non-veterans, OR 1.54, 95% CI 1.01–2.35, *p* = 0.045. Testing for interaction closely approached statistical significance, *p* = 0.067.

## Discussion

### Main findings

Despite the theoretical increased risk of dementia in veterans based on their known increased risks of cardiovascular disease (Bergman et al., 2014, 2018), PTSD (Iversen et al., 2009) and traumatic/mild traumatic brain injury (Shively & Perl, 2012), reassuringly we find no evidence to support the hypothesis that veterans are at an increased risk of dementia overall in comparison with people who have never served. The veterans in our study were aged up to 73 years at the end of data collection, and therefore emerging differences might have been expected to become apparent. This was not the case. Our findings support those of the systematic review by Peterson, Veazie, Bourne, & Anderson (2020) which also found no overall difference in rates of dementia between veterans and non-veterans. We have confirmed an increased risk of dementia in both veterans and non-veterans with PTSD, but with no significant difference between the two groups in this respect. A history of mood (depressive) disorder was a major risk factor for the subsequent development of dementia in both veterans and non-veterans, but for this the risk was disproportionately increased for veterans.

Our findings differ from those of the recent case-control study by Greenberg et al. (2020), which found no evidence of an increased risk of dementia in association with service-related mental health disorders. However, there are substantial differences between the two studies. The case-control study subjects were older, and only 14% of the dementia cases identified would have met the age criteria for our study; one-third of the dementia cases were National Service (conscription) veterans, who were excluded from our study. The veterans were self-declared and self-identified, whereas those in our population-based study were identified from a national database; nor were we able to distinguish between service-related mental health conditions and those arising from other causes. Furthermore, none of the case-control study participants had less than 2 years' service; in our study, veterans of any length of service were eligible for inclusion, and our other study has shown that the risk of mental health disorder is greatest in those with the shortest service (Bergman et al., 2016). Nonetheless, the findings of the case-control study tend to support the findings of Woodhead et al. (2011) that the mental health of older National Service veterans does not differ from that of the non-veteran population.

As dementia is predominantly a condition of older people, the prevalence has only recently begun to increase in our cohort, of whom the oldest members were aged 73 years at the end of data collection. It is not yet possible to examine an older group of veterans in comparison with a large representative matched cohort of non-veterans since most UK men born earlier are veterans of National Service, which finished in 1962, and the only non-veterans in that age group are those who were medically unfit for military service, or who were working in ‘reserved’ occupations which were predominantly manual in nature. Therefore, the picture of dementia risk in UK veterans is only just beginning to emerge, and may change as the cohort ages.

### Antecedent conditions and comorbidities

Although our results confirm the association between dementia and PTSD, we find the effect may be weaker in veterans. However, the reverse is the case for mood disorder, and although there is a highly significantly increased risk of dementia in both veterans and non-veterans with mood disorder, the effect is much stronger in veterans. Both have implications for the care of older veterans, but it is the association with mood disorder which is likely to have the greatest impact numerically. Recent studies show common mental disorders affecting 23% of veterans of recent operations, compared with 8% for PTSD (Rhead et al., 2020). Even in the relatively small number of dementia cases in our study, there were more than five times as many with a history of mood disorder than PTSD, although disclosure controls preclude giving absolute numbers. It is not yet known whether timely diagnosis and treatment of mood disorder, or indeed PTSD, might mitigate the risk of dementia (Byers & Yaffe, 2011), which would in part depend on whether there is a causal association. Alternatively, it has been postulated that depression and dementia may share common pathways including chronic inflammation (Byers & Yaffe, 2014), and vascular disease. Nonetheless, the clear and well-documented association of these conditions with dementia reinforces the need for identification and management of all mental health disorders, in both the veteran and non-veteran populations, well in advance of old age. The possible misclassification of early dementia as PTSD or depression also warrants note, as does the possibility that these conditions may be the first indication of dementia; healthcare providers should carefully assess the older veteran presenting with recent onset of apparent PTSD or depression for cognitive deficits, in order to ensure access to appropriate management.

### Strengths and limitations

The major strength of this study is that it was based on a large cohort covering the whole of Scotland, including over 4200 veterans with a mental health diagnosis, followed up for up to 37 years and up to 73 years of age. Data were obtained from computerised health records and, therefore, were not dependent on personal recall and subject to recall bias.

Limitations of the study include possible loss to follow-up of subjects due to migration away from Scotland, for which no data are available, and the lack of any follow-up data prior to 1 January 1981. The lower limit of the birth cohort at 1945 means that the number of cases of dementia is still small, and this has limited the statistical power of the study. As a 100% sample of eligible veterans, there was no scope to increase the size of the veteran cohort. Notwithstanding this limitation, as dementia in UK veterans is currently under-researched and has been highlighted as a priority area for research, this preliminary study is the first based on a large national cohort to contribute to filling the knowledge gap. Mental health conditions diagnosed and treated solely in primary care could not be identified; therefore, our data reflect the more severe end of the spectrum of mental health disorders and will underestimate the overall incidence of the condition in the community. We have made the assumption that there is no systematic difference between veterans and non-veterans in the recording of dementia in secondary care and mental healthcare in-patients other than the incidence of the condition, and that a comparison of the risk of cases in this population reflects the relative risk in the community. Reverse causality could not be excluded in the associations between dementia and either PTSD or depressive disorder. We were unable to adjust for a history of traumatic brain injury, and have not adjusted for physical comorbidities; this could be explored in a future study. We studied regular personnel only as Reservists cannot be separately identified and are therefore included with the non-veterans; this may have led to underestimation of any differences between veterans and non-veterans, although the number of Reservist veterans with dementia in our dataset is likely to be small. Data on combat exposure in the veterans were not available, nor did we have information on the service to which a veteran had belonged (Royal Navy, Army or Royal Air Force).

## Data Availability

The study remains in progress and no data are available for sharing.
